# Real‐World Efficacy and Safety of Cuticapil Stem Hair Serum as an Add‐On to Minoxidil in Androgenetic Alopecia: A Prospective Observational Study

**DOI:** 10.1111/jocd.70247

**Published:** 2025-05-19

**Authors:** Rachita Dhurat, Girish R. Kulkarni, Anil Ganjoo, Manjul Agarwal, Pradeep Kumari, Pradyuman Vaidya, Sumit Gupta, Shankar Savant, Rathish Nair, Mahima Agarwal, Pankhuri Agarwal

**Affiliations:** ^1^ RD Clinic Mumbai India; ^2^ Torrent Pharmaceuticals Ltd Ahmedabad India; ^3^ Dr. Ganjoo's Skin and Cosmetology Centre New Delhi India; ^4^ Mira Derm Redefining Skin Care New Delhi India; ^5^ Asia Institute of Hair Transplant Pune India; ^6^ Dr. Vaidys's Skin and Laser Clinic Pune India; ^7^ GNH Excel Medical Centre New Delhi India; ^8^ Rejoice Aesthetic Experts Mumbai India; ^9^ Parmarth Mission Hospital New Delhi India

**Keywords:** androgenic alopecia, Capilia longa, Cressatine, Cuticapil stem, female pattern hair loss, Procapil

## Abstract

**Background:**

Androgenetic alopecia (AGA) is a common age‐related hair loss condition influenced by genetics and androgen activity.

**Aim:**

This study evaluated the safety and efficacy of Cuticapil Stem Hair Serum in improving hair loss in AGA patients.

**Method:**

This multicenter, prospective, observational study assessed the real‐world efficacy and safety of Cuticapil Stem Hair Serum in patients with androgenetic alopecia. A total of 60 subjects were evaluated, with the Cuticapil and Standard of Care groups compared using the Mann–Whitney and Wilcoxon signed‐rank tests. Key assessments included the hair pull test, global photographic analysis, and hair shedding count. No adverse events were reported, reinforcing the serum's favorable safety profile.

**Result:**

The Cuticapil + SoC treatment group consistently showed higher mean ranks across multiple parameters compared to the SoC‐only group. The addition of Cuticapil Serum to SoC significantly reduced hair shedding (*p* = 0.0220). In the Hair Pull Test, the Cuticapil + SoC group had a lower mean rank (26.97) than SoC alone (34.03) (*p* = 0.0196), indicating greater efficacy. Global photography analysis showed a higher mean rank of 36.13 in the Cuticapil + SoC group compared to 24.87 in the SoC group (*p* = 0.0057), further supporting its effectiveness.

**Conclusion:**

Cuticapil Stem Hair Serum, when used in addition to standard care, significantly enhances hair growth and reduces hair fall in patients with mild to moderate Androgenetic Alopecia and Female Pattern Hair Loss. It showed superior results in promoting hair growth, reducing shedding, improving hair density, appearance, and managing hair loss.

## 1 Introduction

Hair loss, also known as alopecia, affects people of all ages and genders and can have negative impacts on both their physical and mental well‐being [1]. Patterned hair loss, including androgenetic alopecia (AGA), is the most common type of hair loss affecting both males and females [2, 3, 4, 5]. Despite the different clinical presentations, the pathogenesis is the same in both genders. The age of alopecia onset is usually the 3rd and 4th decades, but hair loss starts immediately after puberty and continues progressively [6]. Approximately 50% of males experience hair loss by the age of 50, while 25% of females show signs of hair loss by the age of 49, increasing to over 50% by the age of 79 [7, 8, 9]. The human hair cycle comprises five phases: anagen, catagen, telogen, exogen, and kenogen phase. The anagen phase (growth) lasts 2–6 years, where active hair production occurs. The catagen phase (transition) lasts 2–3 weeks, involving follicular regression. The telogen phase (resting) lasts 3–4 months, after which hair detaches from the follicle. Finally, the exogen phase represents the shedding of the detached hair, making way for new growth. The kenogen phase is a transitional period between the telogen (resting) and anagen (growth) phases, where the hair follicle remains empty. Prolongation of this phase can lead to delayed hair regrowth and is considered a key contributor to hair thinning [10]. Typically, 85%–90% of scalp hairs are in anagen, 1%–2% in catagen, and 10%–15% in telogen, with a daily physiological hair fall of 50–100 strands [11].

In Indians, a prevalence of 58% in males aged 30–50 years was found. Females have a higher propensity for developing FPHL after menopause because of compensatory adrenal overdrive [1]. Current treatment modalities for hair loss, such as topical minoxidil, oral finasteride, and surgical hair transplantation, have shown varying degrees of efficacy. Minoxidil primarily promotes hair regrowth by prolonging the anagen phase, while finasteride reduces androgen‐mediated follicular miniaturization [12]. However, these treatments often present limitations, including incomplete hair regrowth, slow onset of action, and potential side effects such as scalp irritation or hormonal imbalances [13]. Surgical options are invasive, costly, and not universally accessible. These shortfalls highlight an unmet therapeutic gap, necessitating the exploration of alternative, safer, and more effective options, particularly those leveraging plant‐based or novel formulations to address underlying causes and enhance hair health comprehensively.

With the new research approaches, many new etiologies of hair fall are being determined [14, 15]. As a result, using a well‐formulated topical cosmetic with a multitargeted approach may enhance hair loss management and deliver quicker results. Additionally, many individuals prefer natural extracts due to their lower risk of side effects and better compliance [16].

The Hair Serum (investigational product) used in this study is Cuticapil Stem Hair Serum. It contains natural active ingredients which include Caffeine Herbasome, Procapil, Capilia Longa, Cressatine, and Ronacare Biotin Plus. Caffeine Herbasome is an innovative formulation system with caffeine from green coffee beans and niacinamide that efficiently modulates the barrier properties of the epidermis and transports its active ingredients deep into the skin [17]. Caffeine enhances hair shaft elongation, prolongs anagen duration, and stimulates hair matrix keratinocyte proliferation. Moreover, niacinamide enhances hair growth by preventing oxidative stress‐induced cell senescence and premature catagen entry of hair follicles [18]. Procapil is a combination of three powerful plant‐derived components: oleanolic acid from olive leaves, which inhibits the activity of 5‐alpha‐reductase types 1 and 2; apigenin, a citrus fruit‐derived flavonoid that supports blood vessel dilation; and the peptide glycine‐histidine‐lysine, essential for enhancing promatrix metalloproteinase activity and meeting the metabolic needs of hair [19]. Another ingredient, Capilia Longa, derived entirely from *Curcuma longa* (turmeric), promotes hair growth by rejuvenating hair bulbs through epigenetic mechanisms and improving scalp blood circulation, effectively reducing hair loss. Cressatine is an active ingredient derived from the aqueous extract of watercress ( *Nasturtium officinale* ) and Indian cress ( *Tropaeolum majus* ), leaves and shoots, stabilized with plant glycerin. It stimulates hair growth, strengthens roots, supports keratin production, and promotes hair regeneration through the Wnt signaling pathway. Finally, RonaCare Biotin Plus is a special composition consisting of biotin that is designed to provide the optimal biotin concentration into the deeper scalp layers and provide the desired effect on topical application. Biotin is an effective solution for hair health, promoting longer and healthier growth [20]. The objective of this study was to test the efficacy and safety of Cuticapil Stem Hair Serum as an add‐on strategy to the standard of care in patients with mild to moderate Androgenetic Alopecia (AGA) and Female Pattern Hair Loss (FPHL).

## 2 Methods

### 2.1 Ethical Consideration

This investigator‐initiated, prospective, interventional, real‐world evidence clinical study was conducted in compliance with international and national regulatory requirements, including the Declaration of Helsinki (2013), Good Clinical Practice (GCP) guidelines, and the International Conference on Harmonization (ICH) E6 (R2) guideline. The study also adhered to the Indian regulatory framework, including the New Drugs and Clinical Trials Rules (2019) and the Indian Council of Medical Research's (ICMR) national ethical guidelines for biomedical and health research involving human participants (2017). Moreover, all the patients provided written informed consent before getting enrolled in the study.

### 2.2 Study Design and Population

This multicenter, prospective, observational study was designed to evaluate the real‐world efficacy and safety of Cuticapil Stem Hair Serum in patients with androgenetic alopecia, a common condition characterized by patterned hair loss, in both male and female populations in India. The study employed a parallel‐arm design and was conducted across seven clinical sites in Delhi (*n* = 3), Mumbai (*n* = 2), and Pune (*n* = 2) to ensure a diverse and representative cohort of 60 patients. This multicenter approach facilitated the collection of robust, generalizable data, enhancing the study's external validity. The study enrolled male and female patients aged 18 years or older with a diagnosis of hair loss, characterized by hair shedding for more than 6 months. Eligible participants had a clinical diagnosis of male androgenetic alopecia (AGA) Grade III, IV, or V, or female pattern hair loss (FPHL) Grade II or III. This included both naïve cases and those previously treated with 2% Minoxidil. Patients with no known allergies were included and were required to avoid using hair dye, oil treatments, spa treatments, perming, or straightening during the treatment or observation period. Additionally, participants agreed to exclusively use a nonketoconazole‐based shampoo (Triclenz) throughout the study duration.

The study excluded patients who had undergone cosmetic hair treatments within 1 month prior to enrollment, those with hemoglobin levels below 10.5 g/dL, and individuals with thyroid disorders or systemic conditions such as type 2 diabetes mellitus (T2DM), hypertension, HIV, hepatitis, anemia, heart disorders, cancer (including those undergoing chemotherapy), depression, or psychiatric disorders. Patients taking medications known to cause alopecia were also excluded. Pregnant or lactating females, patients with scalp diseases, heavy smokers, heavy drinkers, and individuals following crash diets were not included in the study.

The primary objectives of this 12‐week study were to evaluate the efficacy of cuticapil stem hair serum in:

1. Reducing hair shedding count after shampooing, as measured from baseline to 12 weeks.
2. Increasing hair count, as assessed through the pull test, from baseline to 12 weeks.
3. Improving global photographic assessment outcomes at 12 weeks.

Secondary endpoints included:

1. Safety assessment of Cuticapil Stem Hair Serum at each study visit.
2. Evaluation of its efficacy as an adjunctive therapy to Standard of Care (Minoxidil Lotion) in patients with androgenetic alopecia (AGA).
3. Patient‐reported outcomes, as measured by the Patient Global Assessment.

### 2.3 Procedures

The study, conducted from August 2023 to July 2024, spanned a total duration of 12 weeks. It involved patients with hair loss who were prescribed a hair serum as an add‐on therapy to the standard of care (topical minoxidil lotion). Baseline assessments included the clinical diagnosis of male and female alopecia (trichoscopic hair diameter diversity > 20%), the collection of demographic data, identification of exacerbating factors, medical history, location of hair fall/hair loss, hair shedding count before shampooing (for females), hair pull test (falling out of > 6 hair strands), global photographic impression, and blinded person photographic impression for scalp hair fall/loss. Photographic impressions were evaluated using a standardized seven‐point rating scale of hair growth compared to baseline (−3: greatly decreased; −2: moderately decreased; −1: slightly decreased; 0: no change; +1: slightly increased; +2: moderately increased; +3: greatly increased) [21].

Follow‐up visit was conducted at the 12th week to reassess hair count before shampooing (for females), hair pull test (falling out of > 6 hair strands) and photographic assessments for scalp hair fall/loss. Safety assessments were performed at the end‐of‐study visit to monitor adverse events and treatment tolerability. Additionally, Patient Global Assessment (PGA) scores were evaluated to assess patient‐reported outcomes. Treatment adherence was monitored using case record forms (CRFs). Patients who completed the full 12‐week treatment period and attended all scheduled follow‐up visits were included in the per‐protocol analysis.

### 2.4 Treatment Allocation

Patients were randomly assigned to either Cuticapil Stem Hair Serum with the standard of care or standard of care alone. Cuticapil Stem Hair Serum consists of (Ingredients: Caffiene Herbasome, Zemea Propandiol, Procapil, DC 193 C, Capilia Longa, Euxyl PE 9010, Cressatine and Ronacare Biotin Plus). The application involved spreading 1 mL of serum evenly over the affected hair loss area and gently massaging it with the fingertips. Both the serum and lotion were to be applied twice daily.

Standard of Care (2% minoxidil lotion alone), to be applied topically on the scalp twice daily.

Patients in both arms were instructed to use Triclenz shampoo as their designated hair cleanser throughout the study.

### 2.5 Statistical Analysis

**FIGURE 1:**
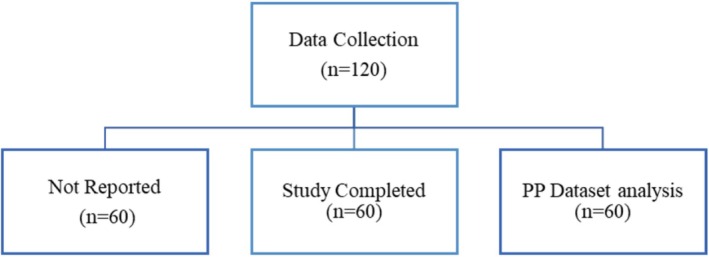
Patients disposition chart.

A total of 60 subjects were included in the statistical analysis. For hair count shedding, a Paired Sample *t*‐test was used to compare baseline and Week 12 data, and an independent sample *t*‐test for group comparisons. Ordinal parameters containing hair pull test, photographic impression, blinded evaluator assessment, global photography, and PGA scores were analyzed using the Wilcoxon signed‐rank test for baseline vs. postbaseline comparisons and the Mann–Whitney test for group comparisons (Figure 1).

## 3 Results

### 3.1 Baseline Patient Characteristics

This investigator‐initiated, real‐world assessment included 60 patients with patterned hair loss. Of these, 30 patients (23 females and 7 males) were prescribed Cuticapil Stem Hair Serum as an add‐on to standard of care, while the remaining 30 patients (19 females and 11 males) received only standard of care. The mean age of the study population was 34.18 ± 11.19 years, mean weight was 64.67 ± 13.41 kg, and the mean height was 159.70 ± 11.16. The majority of males (76.92%) had Grade III AGA, while the majority of females (84.75%) had a Grade II FPHL. Stress was identified as the most common exacerbating factor, affecting 51.76% of participants. Baseline characteristics and patient demographics are detailed in Table 1.

**TABLE 1:** Patient demographics and baseline characteristics.

Variable		*N* = 85
Demographics	Age (years) (mean, SD)	34.18 (11.19)
	Height (cm) (mean, SD)	159.70 (11.16)
	Weight (kg) (mean, SD)	64.67 (13.41)
	BMI (kg)/m^2^ (mean, SD)	25.49 (5.54)
	Gender (*n*, %)	Male: 18 (30.00%)
		Female: 42 (70.00%)

Patient history		Count (*n*)	Percentage (%)
Family history of Alopecia	No	38	63.33%
	Yes	22	36.67%
Exacerbating factors	Stress	35	58.33%
	Poor diet	26	43.33%
	None	11	18.33%
	Medical conditions	1	1.67%
Other medical history	None	56	93.33%
	Thyroid disorders	2	3.33%
	Allergies	2	3.33%

Existing medical history			Count (*n*)	Percentage (%)
Clinical diagnosis of male AGA (trichoscopic hair diameter diversity > 20%)	Gender: Male (*N* = 26)	Grade III	13	72.22%
		Grade IV	4	22.22%
		Grade V	1	5.56%
Clinical diagnosis of FPHL (trichoscopic hair diameter diversity > 20%)	Gender: Female (*N* = 59)	Grade II	36	85.71%
		Grade III	9	21.43%
Location of hair fall/hair loss		Frontal scalp	72	51
		Vertex	37	27
		Temporal scalp	13	9
		Occipital scalp	4	2
		None	1	1
Previous treatment history		Treatment Naïve	60	70.59%

Abbreviations: AGA, androgenic alopecia; BMI, body mass index; FPHL, female pattern hair loss; SD, standard deviation.

### 3.2 Hair Shedding Count (Only Females)

**FIGURE 2:**
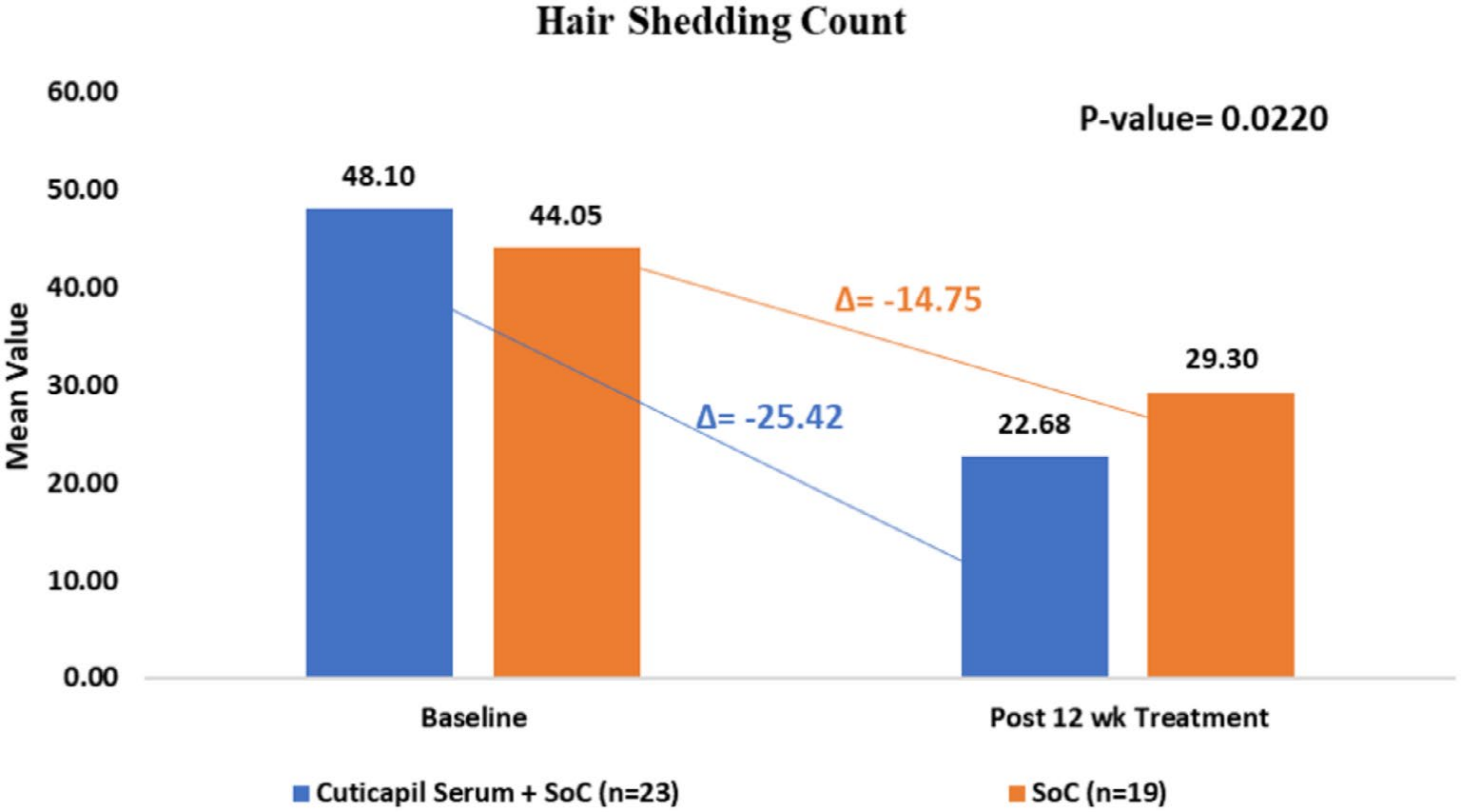
Improvement in hair shedding count from baseline to 12 weeks of treatment.

Hair shedding was assessed at baseline and after 12 weeks of treatment to evaluate the efficacy of both treatment regimens. In the Cuticapil Stem Hair Serum plus Standard of Care (SoC) group, the mean hair shedding count significantly decreased from 48.10 ± 25.61 hairs at baseline to 22.68 ± 17.46 hairs after 12 weeks (mean change: −25.42 hairs, *p* < 0.0001). Similarly, in the Standard of Care group, the mean hair shedding count decreased from 44.05 ± 24.23 hairs at baseline to 29.30 ± 22.29 hairs after 12 weeks (mean change: −14.75 hairs, *p* < 0.0001). A between‐group comparison revealed that the addition of Cuticapil Serum to the SoC resulted in a significantly greater reduction in hair shedding compared to SoC alone (*p* = 0.0220). These findings suggest that Cuticapil Stem Hair Serum enhances the clinical efficacy of the Standard of Care in reducing hair shedding (Figure 2).

### 3.3 Hair Pull Test

**FIGURE 3:**
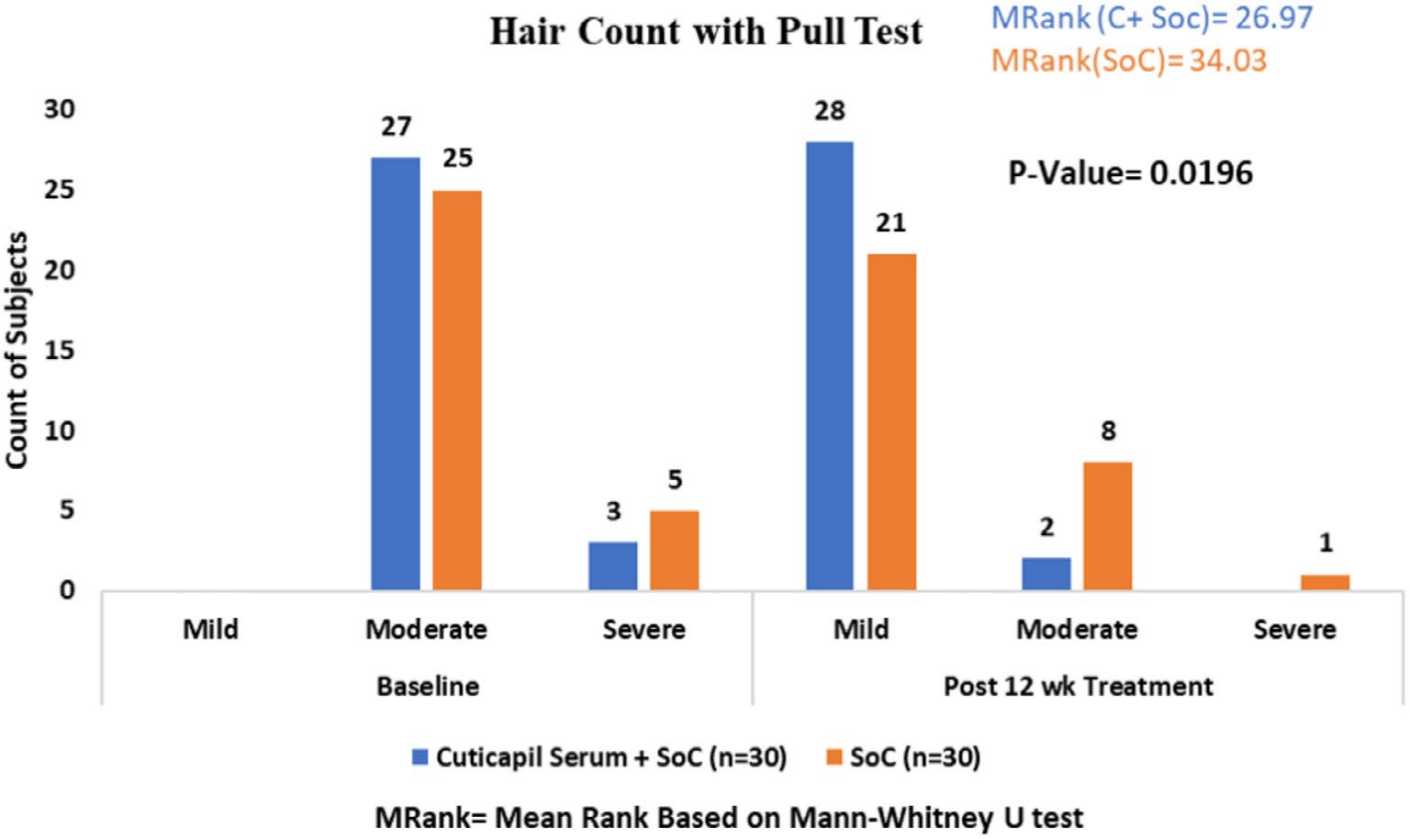
Improvement in hair pull test from baseline to 12 weeks of treatment.

The Cuticapil + SoC treatment group demonstrated a lower mean rank of 26.97 compared with the SoC group of 34.03 in the Hair Pull Test parameter. The statistically significant *p* value of 0.0196 indicates that the combination of Cuticapil and SoC was more effective than SoC alone. A lower mean rank corresponds to greater improvement, reinforcing the superior efficacy of Cuticapil + SoC in reducing hair shedding (Figure 3).

### 3.4 Trichoscopic Photographic Impression

**FIGURE 4:**
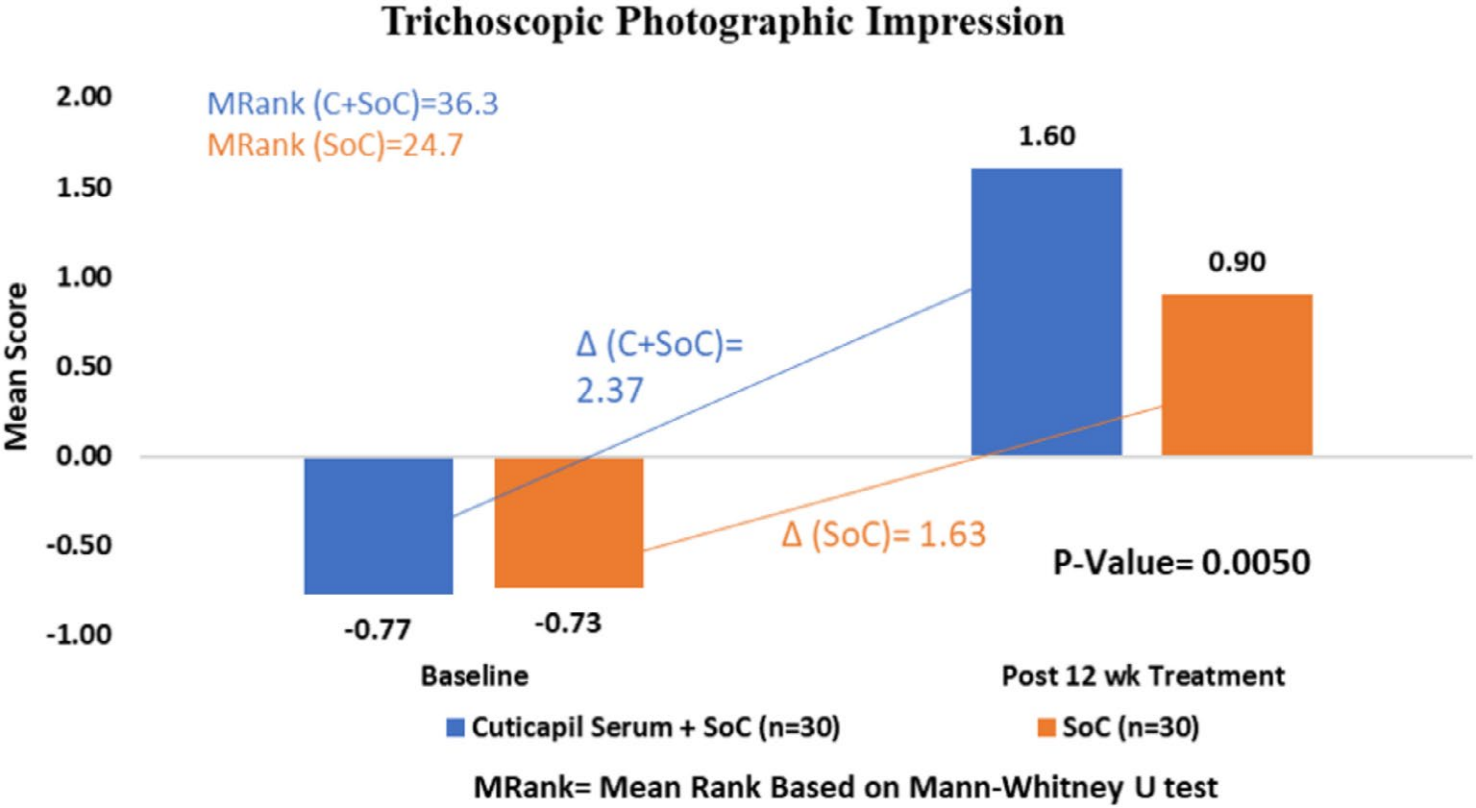
Improvement in photographic impression from baseline to 12 weeks of treatment.

The Cuticapil + SoC treatment group demonstrated a higher mean rank of 36.3 compared with the SoC group of 24.7 in the photographic impression parameter. The statistically significant *p* value of 0.005 indicates that the combination of Cuticapil and SoC was significantly more effective than SoC alone. As a higher mean rank corresponds to greater improvement based on the scoring interpretation, these findings suggest that the addition of Cuticapil to SoC leads to a more noticeable enhancement in hair appearance and overall treatment efficacy, as assessed through photographic evaluation (Figure 4).

### 3.5 Blinded Person Photographic Impression

**FIGURE 5:**
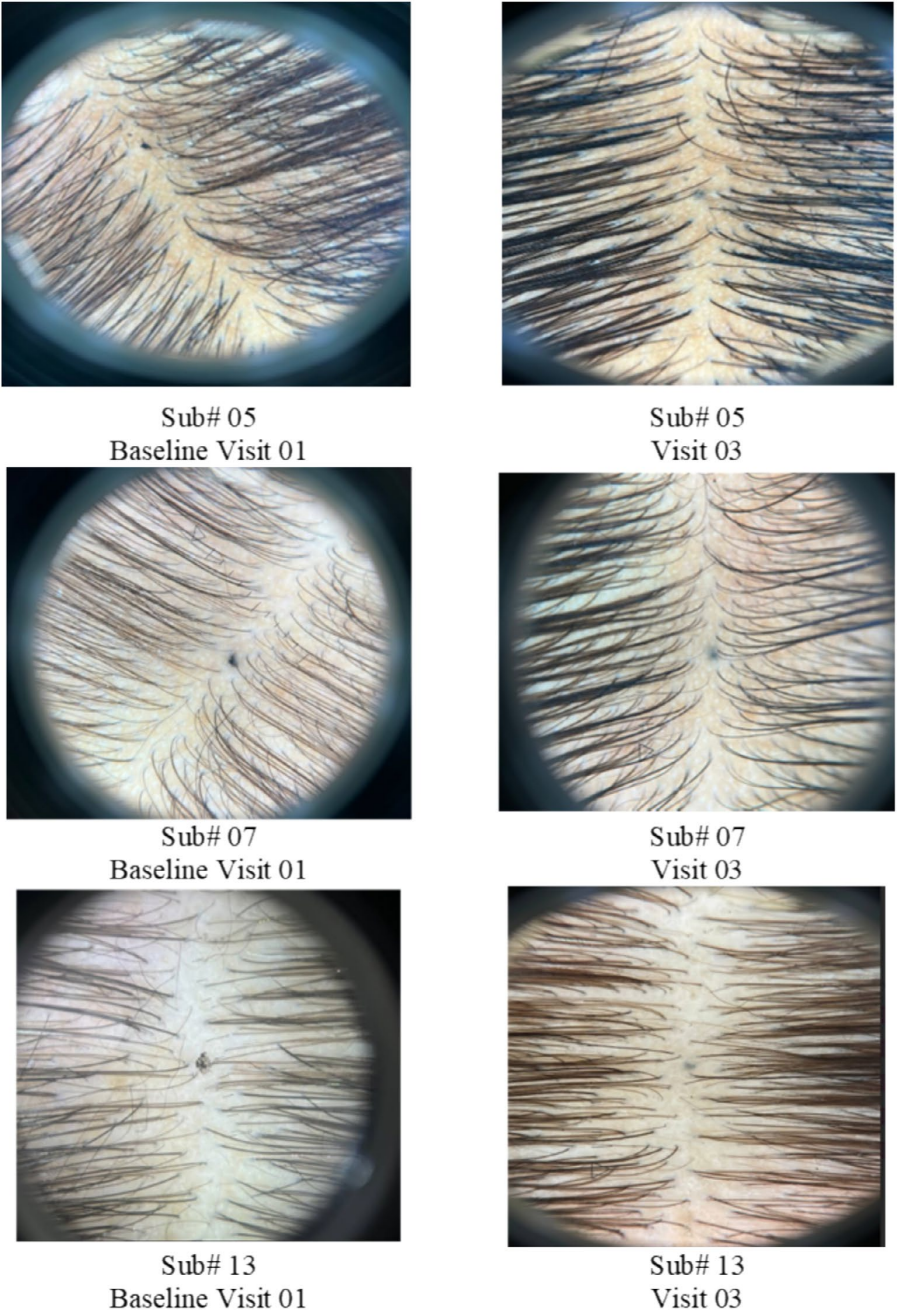
Visible improvement in photographic impression from baseline to 12 weeks of treatment.

**FIGURE 6:**
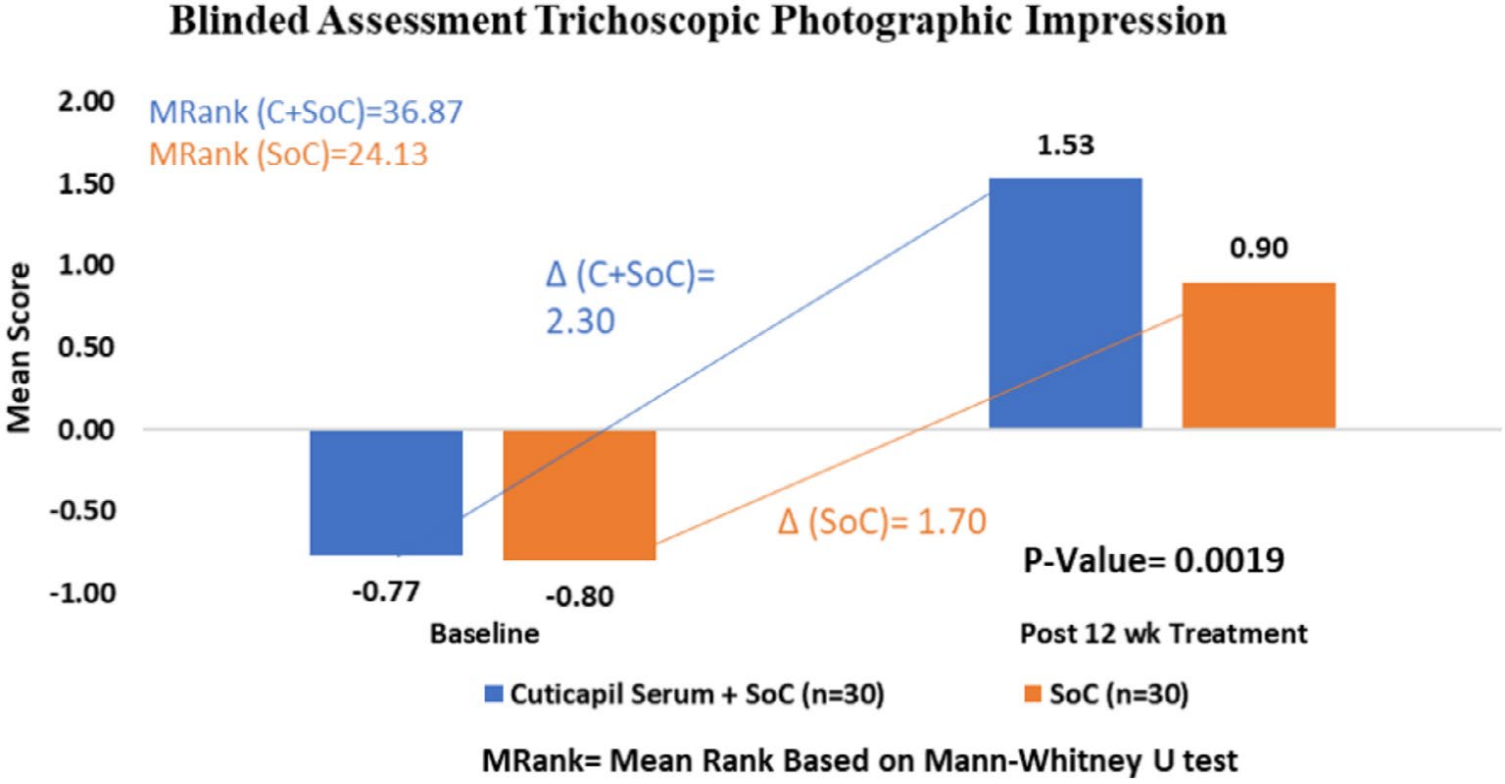
Improvement in blinded evaluator photographic impression from baseline to 12 weeks of treatment.

The Cuticapil + SoC treatment group exhibited a higher mean rank of 36.87 compared to the SoC group of 24.13 in the blinded assessment parameter. The statistically significant *p* value of 0.0019 indicates that the combination of Cuticapil and SoC was significantly more effective than SoC alone. Since a higher mean rank corresponds to greater improvement based on the scoring interpretation, these results suggest that the addition of Cuticapil to SoC led to a more pronounced enhancement in hair health and appearance, as objectively evaluated by blinded assessors (Figures 5 and 6).

### 3.6 Global Photographic Assessment

**FIGURE 7:**
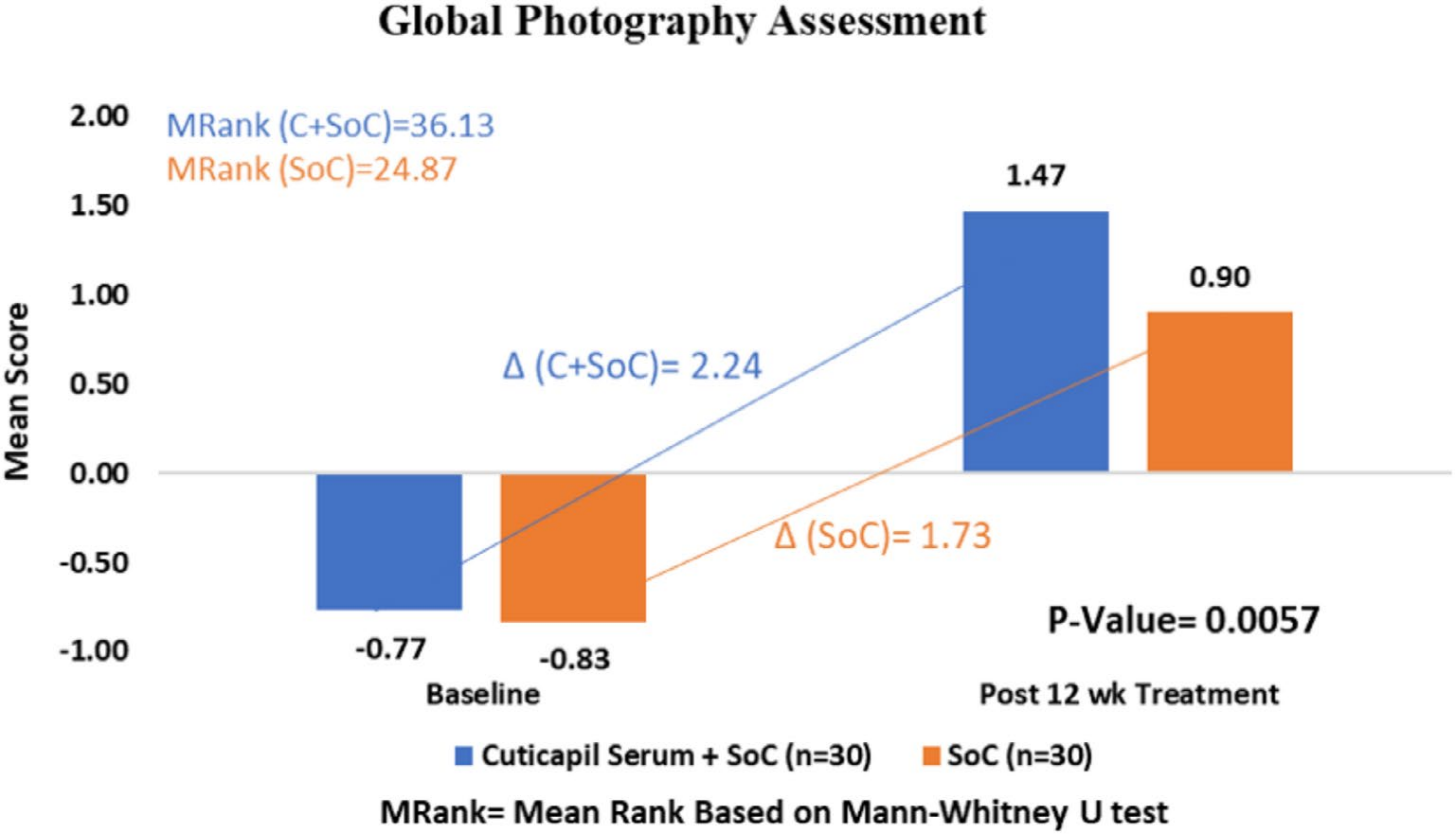
Improvement in global photography from baseline to 12 weeks of treatment.

**FIGURE 8:**
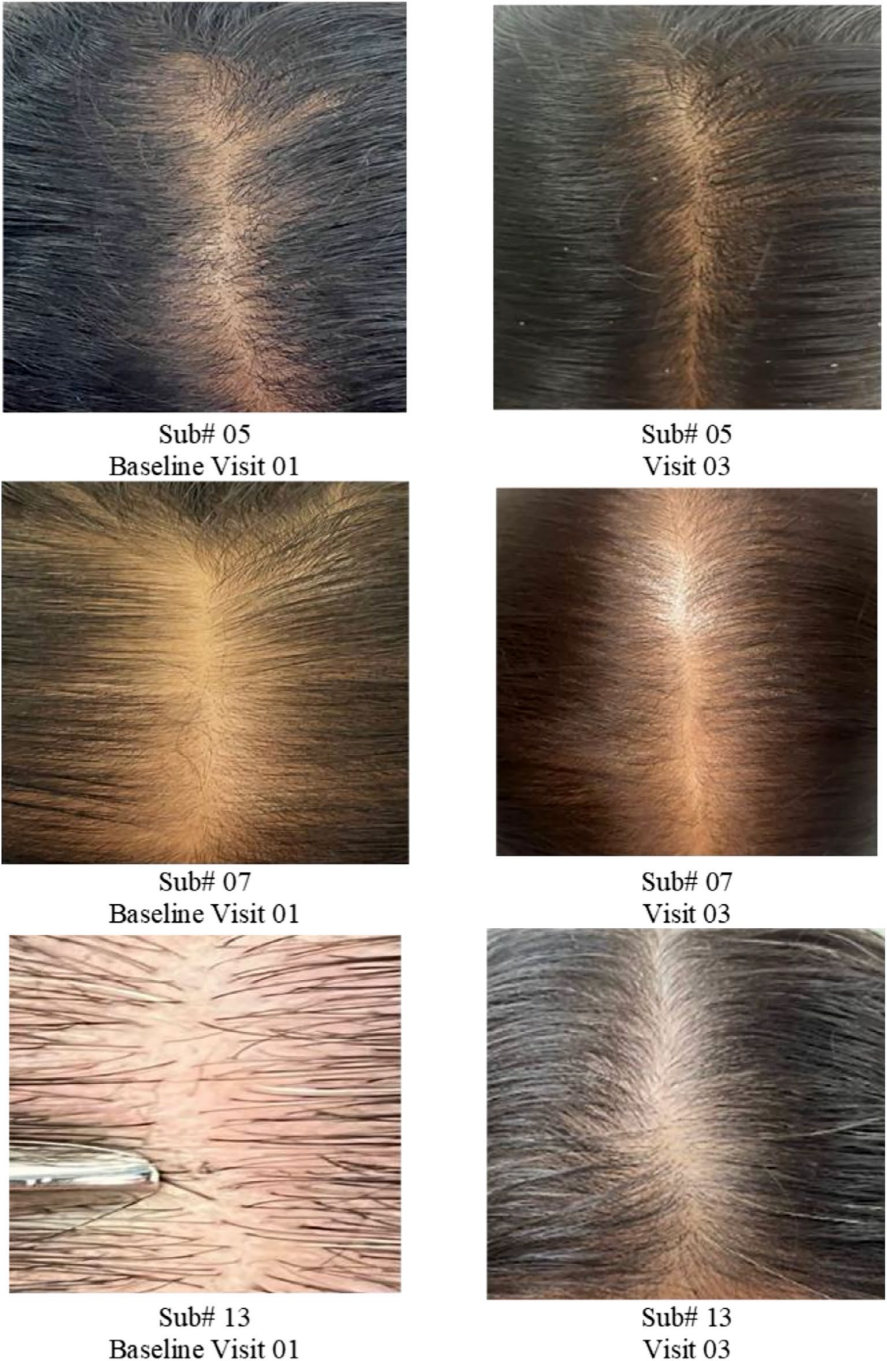
Visible improvement in global photography from baseline to 12 weeks of treatment.

The Cuticapil + SoC treatment group demonstrated a higher mean rank of 36.13 compared with the SoC group of 24.87 in the global photography parameter. The statistically significant *p* value of 0.0057 indicates that the combination of Cuticapil and SoC was significantly more effective than SoC alone. As a higher mean rank reflects greater improvement based on the scoring interpretation, these findings suggest that the addition of Cuticapil to SoC led to more noticeable enhancements in hair density and overall appearance, as captured through global photographic assessments (Figures 7 and 8).

### 3.7 Patient Global Assessment

**FIGURE 9:**
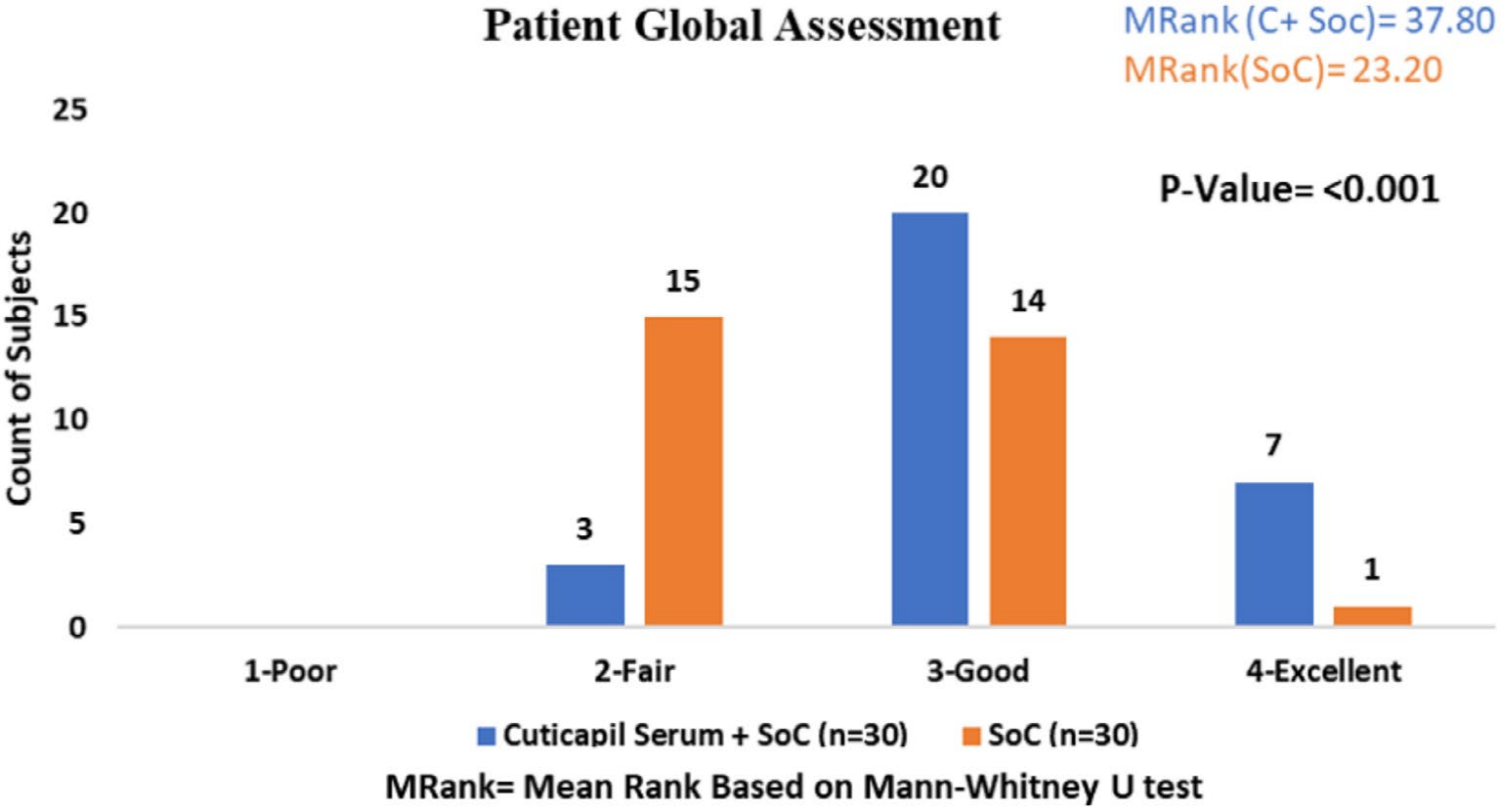
Patient global assessment.

The Cuticapil + SoC treatment group exhibited a higher mean rank of 37.80 compared to the SoC group of 23.20 in the PGA (Physician Global Assessment) parameter. The highly statistically significant *p* value of < 0.001 indicates a strong difference between the groups, suggesting that the combination of Cuticapil and SoC was substantially more effective than SoC alone. Since a higher mean rank corresponds to greater improvement based on the scoring interpretation, these results highlight the superior efficacy of Cuticapil + SoC in enhancing overall hair health and treatment outcomes, as evaluated by physicians (Figure 9).

### 3.8 Positive Hair Pull Test

**FIGURE 10:**
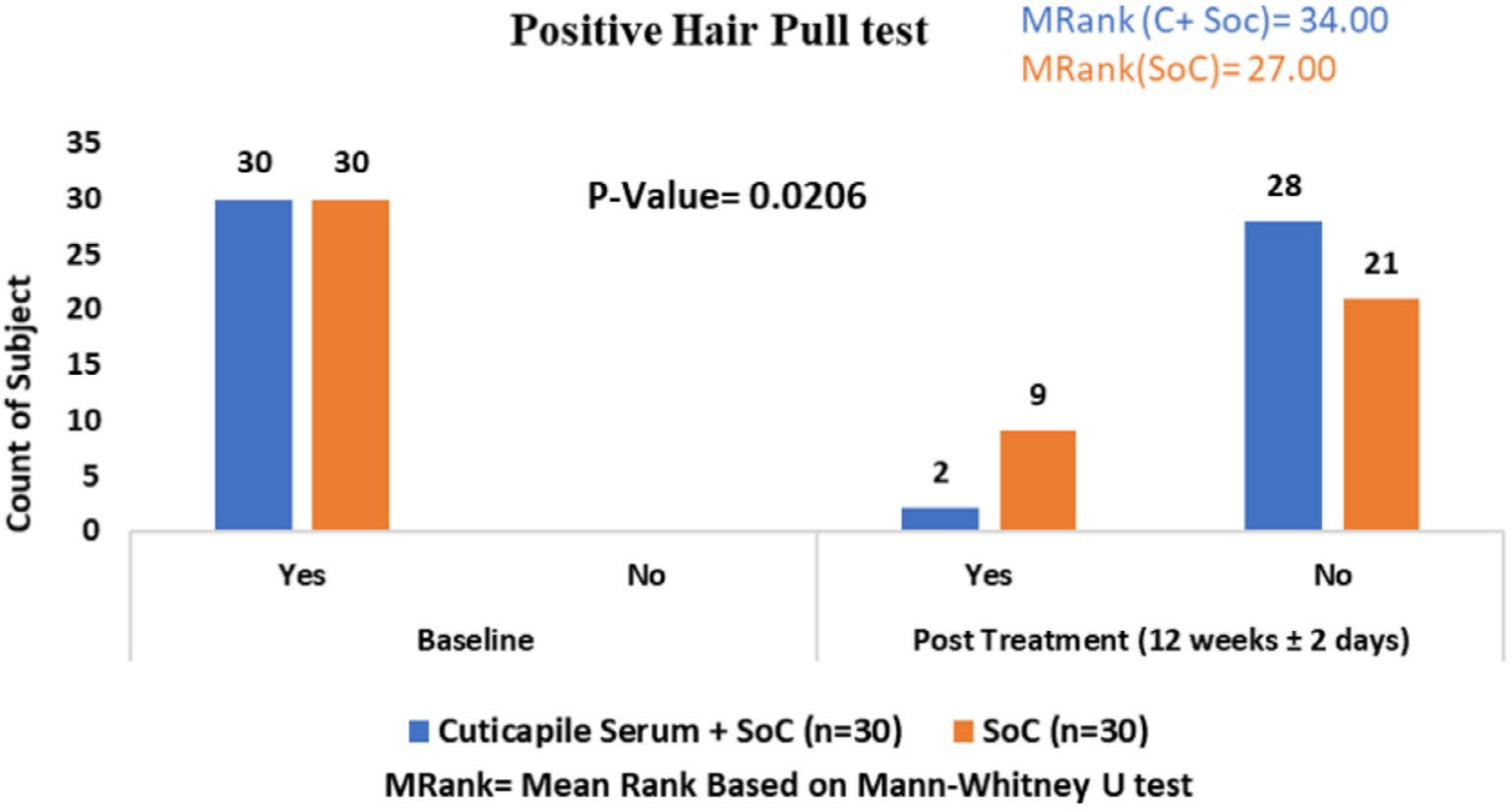
Positive hair pull test.

The Cuticapil + SoC treatment group had a higher mean rank of 34.00 compared with the SoC group 27.00 in the positive hair pull test parameter. The statistically significant *p* value (0.0206) indicates that the combination of Cuticapil and SoC was notably more effective than SoC alone. As a higher mean rank reflects greater improvement based on the scoring interpretation, these findings suggest that adding Cuticapil to SoC led to a more significant reduction in hair shedding, demonstrating its enhanced efficacy in strengthening hair and reducing hair loss (Figure 10).

### 3.9 Safety Assessment

The safety assessment Cuticapil Serum with SoC demonstrated excellent tolerability throughout the study. All patients reported no adverse events, highlighting the strong safety profile. Furthermore, no serious adverse events (SAEs) were observed, reinforcing the treatment's exceptional safety and tolerability.

These findings demonstrate that the addition of Cuticapil Serum with SoC is a well‐tolerated and safe treatment option for the long‐term management of androgenetic alopecia, with a minimal risk of adverse effects.

## 4 Discussion

Androgenetic alopecia (AGA), commonly known as male or female pattern baldness, is the most common type of hair loss, affecting both men and women, with a higher prevalence in men [22]. AGA is influenced by genetic and hormonal factors, particularly the effects of androgens (male hormones), which shorten the hair growth phase and cause hair follicles to shrink. In men, it typically results in receding hairlines and bald spots on the crown, while women usually experience diffuse thinning, especially along the parting of the hair [23].

Our study aligns with previous findings demonstrating the efficacy of key bioactive ingredients in promoting hair growth and counteracting androgen‐mediated hair loss. The in vitro study by Fischer et al. highlighted caffeine as a potent stimulator of hair follicle growth, effectively counteracting testosterone's suppressive effects and enhancing hair shaft elongation. Similarly, our study demonstrated that Cuticapil Stem Hair Serum, containing Caffeine Herbasome, contributed to significant improvements in hair shedding and density [24, 25]. Additionally, research on niacinamide suggests its role in extending the anagen phase by preventing premature catagen entry and cellular senescence through downregulation of DKK‐1, p16, and p21. This aligns with the observed efficacy of our serum, which includes Procapil and Capilia Longa, known for supporting follicular health and hair regeneration, further reinforcing the potential of bioactive formulations in androgenetic alopecia (AGA) management [18].

Cressatine is an active ingredient derived from the aqueous extract of watercress ( *Nasturtium officinale* ) and Indian cress ( *Tropaeolum majus* ), leaves and shoots. *Tropaeolum majus* is a rich source of phytochemicals, and it has been used since a long time as an antiseptic, purgative, hair tonic, and anti‐inflammatory agent [26]. *Nasturtium officinale* extract promotes hair growth, strengthens hair, prevents hair fall, increases hair length and density [27, 28], and has antioxidant effects [29].

Our study findings further support the efficacy of Procapil, a key ingredient in Cuticapil Stem Hair Serum, in promoting hair growth and reducing hair shedding in androgenetic alopecia (AGA). A randomized study on 54 patients demonstrated that a combination of Procapil with Platelet‐Rich Plasma (PRP) therapy resulted in an 11.9% improvement from baseline over 6 months. While the Redensyl, saw palmetto, and biotin (RSB) with PRP group showed a greater 21.9% improvement, both groups exhibited significant hair regrowth, highlighting Procapil's role in follicular health. Similarly, our study observed positive outcomes with Cuticapil Stem Hair Serum, which contains Procapil, leading to enhanced hair density, scalp health, and reduced hair shedding. These findings reinforce the potential of bioactive formulations like Procapil in AGA treatment, either as monotherapy or in combination with other growth‐stimulating agents [19].

A randomized study was conducted by Karaca et al. including 120 patients from 18 to 55 years of age to compare the efficacy of topical 2% minoxidil and a topical preparation of the combination of Redensyl, Capixyl, and Procapil (RCP) for the treatment of AGA. The results of the study showed that the topical preparation of RCP was 64.7% efficacious against AGA compared to the 2% minoxidil group, as assessed by the researcher evaluation score. Hair recovery in the RCP group was found to be 2.54 times higher than that in the minoxidil group at the end of 24 weeks of treatment [30]. Our study aligns with both these studies where an improvement in hair growth had been seen after the usage of the topical preparation.

Moreover, Ronacare Biotin Plus, which contains biotin, maintains normal skin, hair, and normal immune function and helps facilitate healthy growth and strengthening of hairs [31, 32, 33]. Patel et al. conducted a comprehensive review on the use of biotin for hair loss and identified 10 reports with a diagnosis of alopecia in the literature that consistently reported improvements in hair growth upon biotin supplementation [20]. A randomized, double‐blind, placebo‐controlled study was conducted to evaluate the impact of an oral supplement containing biotin (as one of the main ingredients) on hair growth and shedding in women with self‐perceived thinning hair. After a 90‐day supplementation period, the study revealed a reduction in hair shedding, enhancements in hair growth, strength, and overall hair health [34].

Currently, there is no permanent cure for AGA, which often causes anxiety and depression in patients. The US FDA has approved two treatments: topical minoxidil and oral finasteride [35]. The most common side effect of topical minoxidil is irritant contact dermatitis, which is characterized by symptoms such as scaling and itching. In women, minoxidil use can lead to facial hypertrichosis [36].

In our clinical study on androgenetic alopecia, we evaluated the synergistic effects of a combination of caffeine, Procapil, Capilia Longa, and Cressatine on hair growth. Caffeine stimulates hair follicles and enhances scalp circulation, while Procapil strengthens hair follicles and inhibits dihydrotestosterone (DHT). Capilia Longa, with its antioxidant and anti‐inflammatory properties, supports follicle health and promotes growth. Cressatine nourishes follicles and encourages regeneration. Collectively, these ingredients work synergistically to reduce hair thinning and promote healthier hair growth.

This clinical study was performed in real‐world settings in seven centers across India. The study successfully met its predetermined objectives, demonstrating improvements in hair shedding count, hair pull test, photographic impression, blinded person photographic impression, global photographic evaluation, PGA scores, and safety assessment following treatment with the Cuticapil Stem Hair Serum with SoC and SoC alone at 12 weeks, as prespecified.

The addition of Cuticapil Stem Hair Serum with SoC showed a significant reduction in hair shedding and improvement in hair density and quality, indicating its potential as an effective intervention for managing androgenetic alopecia. At baseline, both treatments showed moderate to severe hair loss, with patients showing active shedding. After 12 weeks, a marked improvement in hair retention was observed in patients receiving the Cuticapil Stem Hair Serum with SoC, with a substantial percentage of patients achieving mild hair loss. The addition of Cuticapil Stem Hair Serum enhanced the ability of SoC to reduce hair shedding, supporting its role as an adjunctive treatment. Photographic impressions further revealed the effectiveness of the additional therapy, with a greater number of patients in the group achieving moderate to significant improvements in hair quality and appearance. The safety profile of the additional therapy of Cuticapil Stem Hair Serum with SoC was evaluated and found to be favorable. Notably, no serious adverse events (SAEs) occurred during the study, indicating that the addition of Cuticapil Stem Hair Serum to SoC does not appear to significantly enhance the risk of adverse effects, thereby supporting its safety and tolerability in clinical practice.

While our study provides valuable insights into the efficacy and safety of Cuticapil Stem Hair Serum, several limitations should be noted. The relatively small sample size and short duration of the study may limit the generalizability of our findings. Additionally, the study population was limited to patients with mild to moderate AGA and FPHL, which may not reflect the broader population of individuals with more severe forms of hair loss. Future research with larger, more diverse populations and longer follow‐up periods is needed to confirm these results and explore the long‐term effects of the serum.

## 5 Conclusion

The objective of this study was to test the efficacy and safety of Cuticapil Stem Hair Serum as an add‐on strategy to the standard of care in patients with mild to moderate androgenetic alopecia (AGA) and female pattern hair loss (FPHL). Our findings indicate that the serum significantly improves hair growth and reduces hair fall compared to the standard of care alone. Results suggest that Cuticapil Stem Hair Serum could be a valuable and safe addition to current treatment protocols for AGA and FPHL. By enhancing hair growth and reducing hair fall, Cuticapil Stem hair serum positions itself as a valuable complement to existing treatment protocols, offering patients a promising new option for managing hair loss effectively. Overall, this study contributes to the growing body of evidence supporting the use of innovative treatments for hair loss and highlights the potential of Cuticapil Stem Hair Serum in clinical practice.

## Author Contributions

All authors contributed significantly to this study. G.R.K. and R.N. were involved in the conceptualization and design of the study and analyzed the data. R.D., G.R.K., A.G., M.A., P.K., P.V., S.G., S.S., R.N., M.A., and P.A. participated in data collection and interpretation of the results and contributed to the drafting and critical revision of the manuscript. All authors have read and approved the final manuscript.

## Ethics Statement

Prior to study initiation, ethics committee approval was obtained for all the sites from as follows: Park Hospital—Delhi, Ethicare Ethics Committee—Mumbai, Royal Pune Independent Ethics Committee—Pune. The study was registered with the Clinical Trials Registry of India (CTRI/2023/07/055158), ensuring transparency and accountability.

## Conflicts of Interest

Mr. Girish Kulkarni and Dr. Rathish Nair declare employment from Torrent Pharmaceuticals Ltd., Ahmedabad. All other authors declare no conflicts of interest.

## Data Availability

The data that support the findings of this study are available on request from the corresponding author. The data are not publicly available due to privacy or ethical restrictions.
